# Multimodal medical image fusion in NSST domain with structural and spectral features enhancement

**DOI:** 10.1016/j.heliyon.2023.e17334

**Published:** 2023-06-16

**Authors:** Sajid Ullah Khan, Fahim Khan, Shahid Ullah, YoungmoonLee, Sami ulQudoos, Bumshik Lee

**Affiliations:** a Multimedia Information Processing Lab, Department of Information and Communication Engineering, Chosun University, Gwangju, South Korea; b Department of Computer Engineering, Gachon University, Seongnam-si13120, South Korea; c Faculty of Engineering, University Malaysia Sarawak, Malaysia; d Department of Robotics, Hanyang University, Ansan-si 15558, South Korea; e Department of Computer Science, Comsats University, WahCantt, Pakistan

**Keywords:** Multimodal medical image fusion, Structural and spectral features, Principle component analysis, Non-subsampled Shearlet transform

## Abstract

For the past 25 years, medical imaging has been extensively used for clinical diagnosis. The main difficulties in medicine are accurate disease recognition and improved therapy. Using a single imaging modality to diagnose disease is challenging for clinical personnel. In this paper, a novel structural and spectral feature enhancement method in NSST Domain for multimodal medical image fusion (MMIF) is proposed. Initially, the proposed method uses the Intensity, Hue, Saturation (IHS) method to generate two pairs of images. The input images are then decomposed using the Non-Subsampled Shearlet Transform (NSST) method to obtain low frequency and high frequency sub-bands. Next, a proposed Structural Information (SI) fusion strategy is employed to Low Frequency Sub-bands (LFS's). It will enhance the structural (texture, background) information. Then, Principal Component Analysis (PCA) is employed as a fusion rule to High Frequency Sub-bands (HFS's) to obtain the pixel level information. Finally, the fused final image is obtained by employing inverse NSST and IHS. The proposed algorithm was validated using different modalities containing 120 image pairs. The qualitative and quantitative results demonstrated that the algorithm proposed in this research work outperformed numerous state-of-the-art MMIF approaches.

## 1 Introduction

Multimodal medical image fusion (MMIF) has recently become a popular topic in the scientific community [1,2]. Multimodal medical image fusion refers to the fusing of two or more images derived from distinct imaging modalities. The final combined image has detail information than single source image and is useful in clinical diagnosis [3,4]. For example, Positron Emission Tomography (PET) is a pseudo-color image that depicts organic changes such as cancer cells. Magnetic Resonance (MR) is a gray scale image that depicts anatomical structures such as blood flow, soft tissues, tumor, stroke, and location. However, it can't show metabolic information such as cancer cell. As a result, fusion methods help the radiologist diagnose disease more accurately and with less effort.

All MMIF are categorized into three levels: decision level, pixel level, and feature level [5,6]. However, structural and spectral enhancement deals with pixel information. Therefore, this research work relates to pixel level MMIF. Furthermore, to avoid color distortion in the spatial domain, the multi-resolution transform or multi-scale transform (MST) is the most widely used method to overcome color distortion and improve the accuracy of the fused image. MST decomposes the input images into High Frequency Sub-bands (HFS's) and Low Frequency Sub-bands (LFS's). Various fusion strategies are then applied to fuse these coefficients. At the end, inverse transform is applied to generate the final fused image. In the MST domain, the most often used algorithms are Laplacian Pyramid (LP), Non-subsampled Contourlet Transform (NSCT), Wavelet Transform, Discrete Wavelet Transform (DWT), and NSST [7,8]. If proper fusion rules and scale decomposition are not followed, the fused image will have unexpected block issues. To address these issues, a novel IHS in the NSST domain with structural and spectral feature enhancement is proposed. The decomposed LFS's contains texture and background information. As a result, we processed LFS's using the proposed Structural Information (SI) fusion rule. Similarly, HFS's contains the most pixel level information, and we used PCA as a fusion strategy to obtain the complementary information. At the end, the fused image is generated using inverse NSST and IHS. The specific contributions proposed in this research Work are as follows:

a. MMIF model based on IHS in NSST domain
b. The application of proposed SI as a fusion strategy to fused LFS's to enhance the texture and background information
c. The application of PCA as a fusion strategy to fused HFS's to get the complementary pixel level information

The qualitative and quantitative results showed that our proposed method outperformed the comparative image fusion methods.

## 2 Related work

In the literature, various efficient image fusion methods have been described such as HIS, PCA, and Brovey Transform (BT) etc. [9,10]. Although the PCA method is commonly employed in image fusion to extract pixel level information. However, it is incapable of dealing with local vibration of intensity changes. The IHS method initially converts the low spatial resolution RGB image into IHS components. After necessary operations, the IHS image is transformed back to an RGB image. IHS method performs efficiently in terms of color visualization [11]. However, the IHS approach suffers from distortion since the MR image differs from the substituted “I" component of the PET image. HSV method proposed in Ref. [12] fused the MRI and SPECT images by employing a color encoding mechanism. It provides better structural and metabolic features with less spectral distortion. Similarly, a fusion method proposed in Ref. [13] merged the features of IHS with PCA and get acceptable results. The resulting image has high spatial information and less spectral distortion. However, it has an image registration problem. A combination of PCA and SWT method was proposed in Ref. [14] achieves better results in contrast as compared to IHS and ICA. However, all of these techniques have color distortion issues.

Color distortion is most likely resolved using MST methods. Two factors affect fusion performance in MST approaches. The first one is how many scales are used for image decomposition, and the second is the fusion strategy for HLF and LFC sub-bands. The texture and background information are related to LHF, whereas the pixel level information is related to HFC. The detailed amount of information extracted, the better the fusion results. The Contourlet Transform (CT) method was presented in Ref. [15] to get the directional information during the fusion process. But it has a shift-invariant issue due to sampling. The NSCT method was proposed in Ref. [16] to tackle the sampling issue. The NSCT method, which is multi-dimensional, multi-resolution, and shift-invariant, is widely used for image enhancement and image fusion. However, NSCT method suffers with smaller scale decompositions problems. In Ref. [17], an NSST method with multi-dimensional, shift-invariant, and multi-resolution properties was proposed. Furthermore, this method overcomes the size of smaller scale decomposition, making it superior to NSCT. Due to the advantages of Biometric fusion system and palmprint recognition, it is highly important to study about multi-source palmprint fusion systems.

To address this issue [18], presented a fusion approach using tow dimensional discrete cosine transform (2DDCT) to extract the palmprints left and right features. In the method, the discriminative parameters and pre-masking are removed using normalization process. As a result, more discerning parameters can be conserved and retrieved from dual source space using discrimination power analysis (DPA), resulting in improved accuracy. Similarly, an ideal technique was presented in Ref. [19] to overcome the difficulties of computational complexity and storage cost. To resolve the issue between security and verification efficiency, this study presents two cancelable palmprint coding systems, PalmHash Code and PalmPhasor Code. To improve the performance of this method, transposition with perpendicular orientation along with score level fusion are employed. In Ref. [20], an innovative approach for improving feature extraction in applications that process images was developed, and texture patterns functioned effectively to evaluate an image. In this method, a Convoluted Local Energy Oriented Pattern (CLEOP) technique is used to improve the efficiency and extract the features.

Furthermore, the NSST and IHS approaches, as well as some other extensively used methods for medical image fusion, are discussed in detail. The idea presented in Ref. [21] used CNN using GoogleNet architecture to avoid an invasive process of the patient. This method helps the doctor to analyze the CT image in 3D forms without the assistance of Radiologist. This method has achieved higher accuracy for polyps detection. Similarly, in Ref. [22], an algorithm is proposed to detect pixel similarities using Full Binary Tree approach. In this method, the intensity values within the kernel are converted into the binary form and compared the binary pattern of the center one with its 8-negihboring pixels. This method most probability helps in facial verification. This method has higher classification results of 77.4%, 77.98 and 77.94 respectively.

We have also provides some prominent fusion rules applied to coefficients processing such as IFS based cosine similarity and Visualization based fusion rules.

### 2.1 Non-Subsampled Shearlet Transform (NSST)

**Fig. 1:**
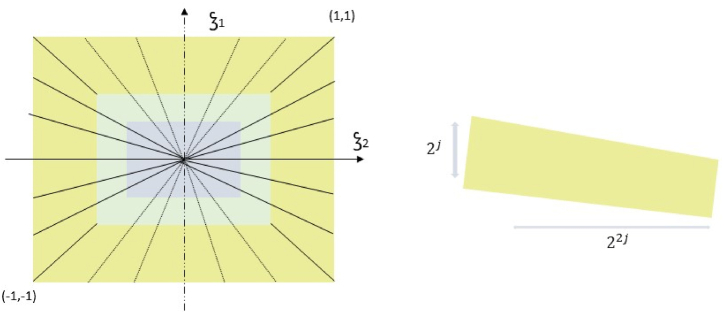
NSST Decomposition (frequency tiling), (a) Frequency plane, (b) Shearlet component size.

The NSST technique is made up of partitioning having multiple scales and directional localization. The input images are decomposed into coefficients using a non-subsampled pyramid approach. It will reduce the image shift sensitivity. The non-subsampled shearing filters are used to decompose the frequency plane into LFS's and many trapezoidal HFS's [17]. The NSST decomposition method is depicted in Fig. 1. The NSST model is expressed in Eqs. (1), (2), (3), (4).

(1) $$A_{MN}\left(\psi \right)=\{\psi_{j,l,k}\left(x\right)={\left|detA\right|}^{\frac{j}{2}}\psi \left(M^{i}A^{j}x-k\right):j,l\in Z,k\in Z^{2}$$

Where *M* and *N* are invertible matrices and |detN|=1.

*M* and *N* can be expressed as:

(2) $$A=\left[\begin{array}{cc} 4 & 0\\ 0 & 2 \end{array}\right],B=$$

Fig. 1 (a) depicts the decomposition (frequency tiling), whereas Fig. 1 (b) shows the Shearlet component ($\psi_{j,l,k})$ size of frequency support. Eqs. (3), (4) shows the NSST and NSST^−1^ functions.

(3) $$\left\{L_{n},H_{n}\right\}=nsst\_decmps\left({Img}_{input}\right)$$

(4) $${Img}_{rev}=nsst\_reconst\left(L_{n},H_{n}\right)$$

Where *nsst_decmps* denotes NSST decomposition for the source image *Img*_*input*_, and *nsst_reconst* denotes NSST reconstruction for the final image *Img*_*rev*_. $L_{n}and\;H_{n}$ are the LFS and HFS respectively.

NSST outperformed the most commonly used MST methods because of its multidimensionality, multi-scale partitioning, and shift-invariant properties. As a result, it's widely used in MMIF.

### 2.2 Intensity, Hue, Saturation (IHS)

**Fig. 2:**
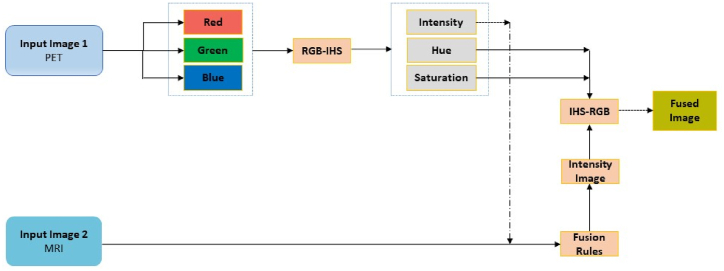
MMIF using HIS.

The IHS method was first used in the field of remote sensing to get complementary information from multispectral and panchromatic images. The IHS method initially converts the low spatial resolution RGB image into IHS components. It begins by converting the RGB channel to IHS components. The intensity component “I" is then substituted with a panchromatic image. Finally, the substitutive panchromatic image is converted back to an RGB image using the unchanged “H" and “S" components of the multispectral image. IHS method performs efficiently in terms of color visualization [11]. Fig. 2 diagrammatically represents PET and MRI source image fusion using the IHS transform method.

### 2.3 Visualization based fusion rules

The contrast of the fused image can be enhanced using visualization based fusion rules. It is calculated by subtracting the grayscale values from the mean values of the image block. Contrast visibility is one of the best examples of this fusion strategy. It can be expressed using Eq. (5).

(5) $$CV=\frac{1}{E\times F}\sum_{\left(e,f\right)\in B_{k}}\frac{\left|f\left(e,f\right)-\mu_{k}\right|}{\mu_{k}}$$

Where *B*_*k*_ is the block dimensionality and $\mu_{k}\text{,}|$ and *E* × *F* are the mean.

### 2.4 IFS based cosine similarity

The IFS function is employed to enhance the features of the fused image by calculating cosine similarity of the input images. It can be expressed using Eq. (6).

(6) $${CS}_{IFS}\left(G,H\right)\frac{1}{n}\sum_{j=1}^{n}\frac{\mu_{G}\left(C_{j}\right)\mu_{H}\left(C_{j}\right)+V_{G}\left(C_{j}\right)V_{H}\left(C_{j}\right)}{\sqrt{\mu_{G}^{2}\left(C_{j}\right)+V_{G}^{2}\left(C_{j}\right)\sqrt{\mu_{H}^{2}\left(C_{j}\right)+V_{H}^{2}\left(C_{j}\right)}}}$$

All these MST methods performed well. However, If proper fusion rules and scale decomposition are not followed, the fused image will have spatial and spectral distortion and unexpected block issues. This research work proposed a novel IHS in the NSST domain with structural and spectral feature enhancement methods to fuse the source image with all complementary information.

## 3 Materials and methods

### 3.1 Data collection

A total of 120 multimodal medical image combinations were used to validate and test the proposed algorithm. We divided these image combinations into four groups such as (1) 22 image combinations of SPECT-MRI and 21 combinations of PET-MRI of Alzheimer's disease, (2) 29 combination of PET-MRI and 18 combinations of SPECT-MRI of glioma disease, (3) 12 combinations of SPECT-MRI and 14 combinations of PET-MRI of hypertensive encephalopathy disease, and (4) 9 combinations of SPECT-MRI and 8 combinations of PET-MRI of normal ageing. These image combinations are downloaded from the Harvard Medical School database named Whole Brain Atlas [23]. Another critical consideration before beginning the fusion process is image registration. It is a critical factor influencing MMIF's performance. As a result, we have registered all pairs with a size of 256 × 256. The datasets may be available on request to the corresponding author.

### 3.2 Proposed method

**Fig. 3:**
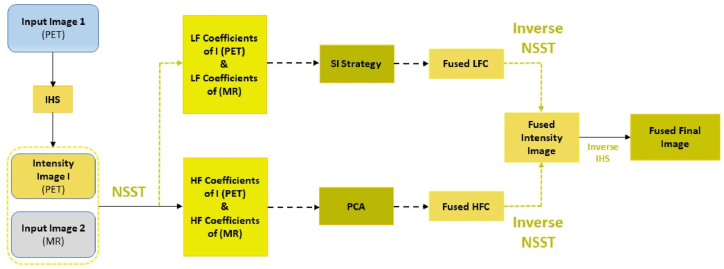
Framework of proposed algorithm.

This section presents the proposed new structural and spectral feature improvement strategy in the NSST Domain for multimodal medical image fusion (MMIF). The proposed architecture is presented in Fig. 3. It consists of four parts: the IHS and NSST decomposition, LFS's and HFS's fusion strategies, and the HIS and NSST reconstruction.

First and foremost, the IHS method is applied to source image 1 (PET image), resulting in a pair consisting of intensity image “I” and source image (MR). The NSST method is then applied to the resulting pair image (intensity I and MR) to obtain the LFS's and HFS's. Section 3.2.1 describes the NSST decomposition process in detail. After that, the LFS's and HFS's of both images (intensity I and MR) are fused using proposed SI and PCA fusion strategies to get the fused Sub-bands/coefficients. Sections 3, 3.2.2.2.3 present the SI and PCA fusion strategies. At the end, inverse NNST and IHS is applied to obtain the final fused image.

#### 3.2.1 NNST decomposition

The NSST decomposition is performed on source images i.e. Intensity image I_PET_ and MR to get the LFS's and HFS's which are ${LFS}_{PET}$, ${HFS}_{PET}^{l,k}$ and ${LFS}_{MR}$, ${HFS}_{MR}^{l,k}$ using Eq. (3). *LFSs* denotes low frequency sub-bands and *HFS*^*l,k*^ denotes high frequency coefficients at *l* level with direction *k*. Eqs. (7), (8) expressed NSST decomposition.

(7) $$\left\{{LFS}_{PET},{HFS}_{PET}^{l,k}\right\}=nsst\_decmps\left(I_{PET}\right)$$

(8) $$\left\{{LFS}_{MR},{HFS}_{MR}^{l,k}\right\}=nsst\_decmps\left(MR\right)$$

#### 3.2.2 LFS fusion using structural information (SI)

The LFS's (${LFS}_{PET}$ and ${LFS}_{MR}$) contain the texture and background details of the input images. These coefficients were fused in the following steps:

i. Initially, we calculated the density of intensity values using Eqs. (9), (10).

(9) $${ID}_{PET}=\mu_{PET}+{Med}_{PET}$$

(10) $${ID}_{MR}=\mu_{MR}+{Med}_{MR}$$

Where *μ* and *Med* denote the mean and median values of *LFS*_*PET*_ and *LFS*_*MR*_. ID is the intensity or pixel information density. Then,

ii. Eq. (11), (12) is used to calculate structural information.

(11) $${SI}_{PET}\left(m,n\right)=\exp\left(\propto \left|{LFS}_{PET}-{ID}_{PET}\right|\right)$$

(12) $${SI}_{MR}\left(m,n\right)=\exp\left(\propto \left|{LFS}_{MR}-{ID}_{MR}\right|\right)$$

Where $\exp\left(\propto \left|{LFS}_{PET}\left(m,n-{ID}_{PET}\right)\right|\right)$ denotes the exponential operator and ∝ represents modulation constraint, respectively.

iii. Weighted mean is applied to obtain the fused LFS's based on Eq. (13).

(13) $$LFS\left(m,n\right)=\frac{{SI}_{PET}\left(m,n\right)\times {LFS}_{PET}\left(m,n\right)+{SI}_{MR}\left(m,n\right)\times {LFS}_{MR}\left(m,n\right)}{{SI}_{PET}\left(m,n\right)+{SI}_{MR}\left(m,n\right)}$$

#### 3.2.1 Principal Component Analysis (PCA)

To get the pixel level information, PCA is employed to HFS's using Eq. (14), (15).

1) Suppose input modalities coefficients are X^1^ and X^2^.

$$A_{1}^{1}A_{1}^{2}$$

(14) $$X1=A_{2}^{1}X2=A_{2}^{2}$$

$$A_{n}^{1}A_{n}^{2}$$

2) Covariance matrix measurements

(15) $$CM\;\left(X^{1},{WX}^{2}\right)=EV\left[\left(X^{1}-\mu_{1}\right)\left(X^{2}-\mu_{2}\right)\right]$$

Where *EV* denotes expectation vector and $\mu_{1},\mu_{2}$ are the coefficients which can be calculated using Eq. (16), (17).

(16) $$\mu_{1}=\frac{1}{n}\sum_{a=1}^{n}X_{i}^{1}$$

(17) $$\mu_{2}=\frac{1}{n}\sum_{a=1}^{n}X_{i}^{2}$$

Then Eigen vectors (Evc) and Eigen values (Evl) are calculated using Eq. (18).

(18) [VvcEvl]=eig(CM)

*Vvc* is calculated to obtain normalized weights using Eq. (19).

(19) $$Ri_{1}=\frac{Vvc\left(1\right)}{\sum \sum Vvc},Ri_{2}=\frac{Vvc\left(2\right)}{\sum \sum Vvc}$$

Finally, Eq. (20) can be employed to calculate the fused coefficient.

(20) $$X_{F}=X^{1}\times Xi_{1}+X^{2}\times Xi_{2}$$

Finally, using Eq. (21), the resulting fused image is obtained by applying inverse NSST and IHS.

(21) $$fusedImg=nsst\_reconst\left(LFC\left(m,n\right),X_{F}\right)$$

## 4 Experimental results

**Fig. 4:**
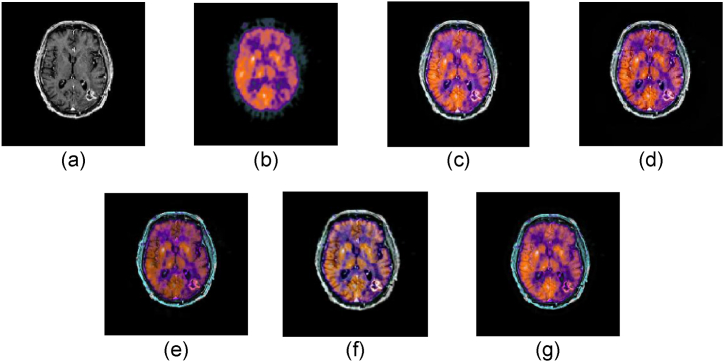
MRI and PET images fusion results of Glioma disease, (a) MRI image, (b) PET image, (c) NSST-PAPCNN [24], (d) CNN [25], (e) CSMCA [26], (f) IFCNN [27], (g) Proposed.

**Fig. 5:**
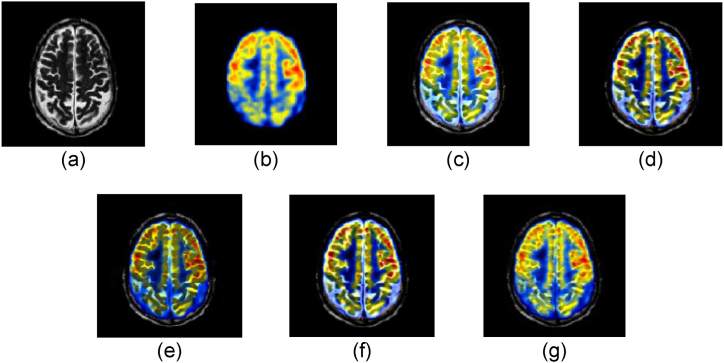
MRI and PET images fusion results of Alzheimer's disease, (a) MRI image, (b) PET image, (c) NSST-PAPCNN [24], (d) CNN [25], (e) CSMCA [26], (f) IFCNN [27], (g) Proposed.

**Fig. 6:**
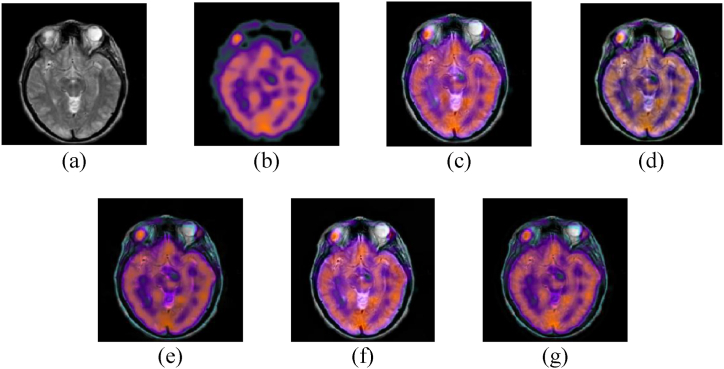
MRI and SPET images fusion results of Hypertensive encephalopathy disease, (a) MRI image, (b) PET image, (c) NSST-PAPCNN [24], (d) CNN [25], (e) CSMCA [26], (f) IFCNN [27], (g) Proposed.

**Fig. 7:**
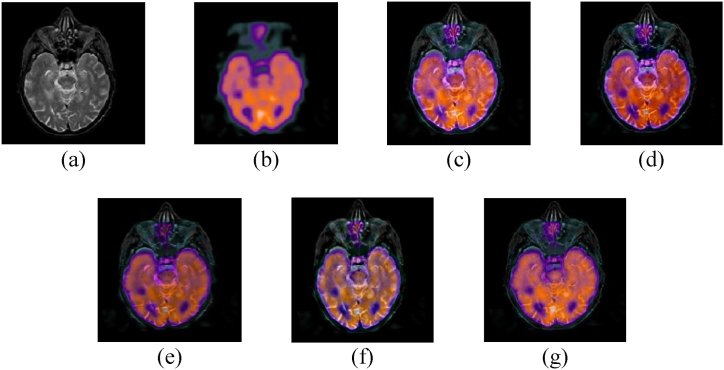
MRI and SPECT images fusion results of Normal aging, (a) MRI image, (b) PET image, (c) NSST-PAPCNN [24], (d) CNN [25], (e) CSMCA [26], (f) IFCNN [27], (g) Proposed.

The performance of our proposed model is discussed in this section. The experiments were carried out with MATLAB 2020a running on a Core i84790 CPU with 1.8 GHz and 16 GB of memory. The experiments were conducted using four datasets: Hypertensive encephalopathy, Alzheimer's, glioma, and normal aging. We compared our model with 4 state of the art MMIF algorithms namely (NSST-PAPCNN [24] S_Link 1), (CNN [25] S_Link 2), (IFCNN [26] S_Link 3), and (CSMCA [27] S_Link 4). The qualitative and quantitative both methods were used to evaluate the accuracy and performance of our proposed model. The source codes for these MMIF methods are available in the supporting material section. We employed the same criteria in our experimental results according to the papers stated above. Fig. 4, Fig. 5, Fig. 6, Fig. 7 depict the subjective evaluation of glioma, Alzheimer's, hypertensive encephalopathy, and diseases using PET-SPECT and PET-MRI images.

According to our observation, NSST-PAPCNN method provided better qualitative results and was near to our proposed algorithm for Glioma disease and normal aging fusion. However, the results are not satisfactory in the case of Alzheimer's and hypertensive encephalopathy diseases. Similarly, the CSMCA model is adequate for the fusion of hypertensive encephalopathy but not for the rest diseases. Furthermore, we cannot rely solely on subjective MMIF algorithm results until there is no loss of structural and spatial features. Therefore, to further evaluate our proposed algorithm, the quantitative results are shown in Table 1, Table 2, Table 3, Table 4, Table 5.

**Table 1:** Quantitative results of Glioma disease (PET-MRI).

	SD	E	SSIM	SC
NSST-PAPCNN [24]	52.8544	3.4341	0.8741	2.9541
CNN [25]	52.5874	3.4347	0.4512	3.1021
CSMCA [26]	**53.9841**	3.4309	0.6521	2.9812
IFCNN [27]	53.4521	3.4315	0.5634	2.5478
Proposed	53.8451	**3.4351**	**0.9541**	**3.2145**

**Table 2:** Quantitative results of Glioma disease (SPECT-MRI).

	SD	E	SSIM	SC
NSST-PAPCNN [24]	**58.5141**	**5.1121**	0.8741	3.1241
CNN [25]	57.8991	5.0021	0.4512	3.4411
CSMCA [26]	58.8745	5.0147	0.6521	3.9812
IFCNN [27]	58.5412	4.9878	0.5634	3.5478
Proposed	58.5051	5.1088	**0.9541**	**4.0145**

**Table 3:** Quantitative results of Alzheimer's disease (PET-MRI).

	SD	E	SSIM	SC
NSST-PAPCNN [24]	**64.2174**	3.1444	0.9214	2.4784
CNN [25]	63.8974	3.0211	0.7845	2.2145
CSMCA [26]	64.1898	3.1145	0.9035	2.4578
IFCNN [27]	64.1424	3.0121	0.8234	2.4122
Proposed	64.1994	**3.1784**	**0.94014**	**2.5123**

**Table 4:** Quantitative results of Hypertensive encephalopathy disease (SPECT-MRI).

	SD	E	SSIM	SC
NSST-PAPCNN [24]	61.4127	4.1784	0.6841	1.2145
CNN [25]	61.2145	4.8541	0.6354	1.1954
CSMCA [26]	**61.8218**	4.7784	0.6751	1.2187
IFCNN [27]	61.1424	4.7499	0.6554	1.2001
Proposed	61.7951	**4.8841**	**0.6921**	**1.2196**

**Table 5:** Quantitative results of Normal aging (SPECT-MRI).

	SD	E	SSIM	SC
NSST-PAPCNN [24]	52.4144	4.5421	**0.7114**	1.2145
CNN [25]	52.2154	4.1245	0.7087	1.1954
CSMCA [26]	**52.8412**	4.7184	0.7102	1.2187
IFCNN [27]	52.3214	4.2499	0.7071	1.2001
Proposed	52.8409	**4.7421**	0.7110	**1.2196**

The performance evaluation metrics used in this research work are Standard Deviation (SD) to compute the intensity variation of the fused image, Entropy (E) to compute the amount of information in a fused image, Structure Similarity Index Measure (SSIM) to compute the similarities between original and fused image, and Structural Content (SC) to compute the strength and structural information of the fused image.

**Fig. 8:**
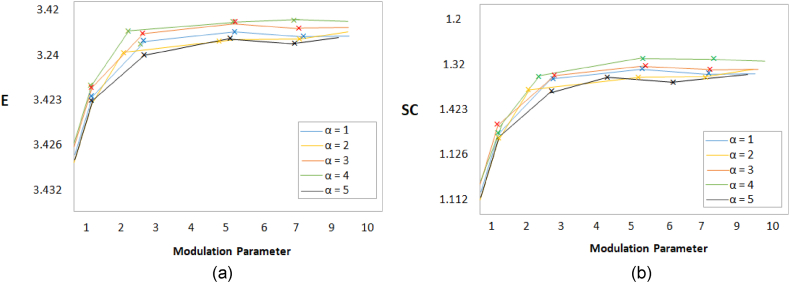
MRI and PET images fusion results, (a) Entropy, (b) Structural contents.

**Fig. 9:**
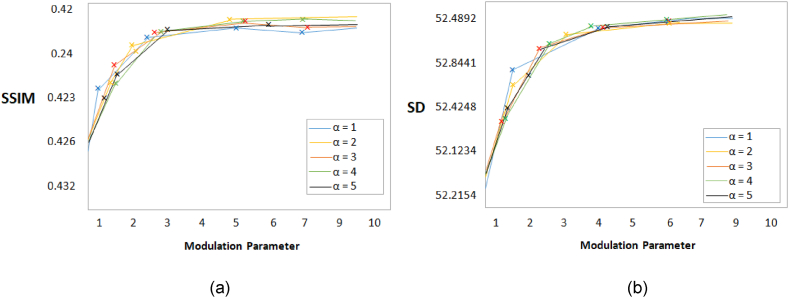
MRI and PET images fusion results, (a) SSIM, (b) SD.

The quantitative results expressed that our proposed algorithm achieved higher results in most of the cases. However, in a few cases, it achieved the second best results. According to our observation, the values for α (modulation parameter) and NSST decomposition scales changed the performance of the proposed algorithm. It provides the best outcomes when α set to 4 and NSST scales set to 3. Fig. 8, Fig. 9 depict these findings.

## 5 Conclusion

This article proposed MMIF algorithm in the NSST domain with structural and spectral feature enhancement. The key advantage of our proposed algorithm is the fusion rules in NSST domain which outclassed all the comparative state of the art methods. The blended NSST decomposition into different scales achieved satisfactory results in MMIF. The objective and subjective results demonstrated that our proposed algorithm outperformed the recent state-of-the-art MMIF methods. Deep learning methods are extensively used in MMIF because they produce good optimised fused image and high intensity variation results. However, it has a number of flaws, including a long data training time, overfitting, and convergence. In the future, we will concentrate on deep learning fusion methods to address the issues of long data training time, overfitting, and convergence, as well as a challenge to improve the region of interest in source images prior to the fusion process.

## Author contribution statement

Sajid Ullah Khan: Wrote the paper and performed the experiments.

Fahim Khan: Conceived and designed the experiments.

Shahid Ullah; Bumshik Lee: Contributed reagents, materials, analysis tools or data.

Youngmoon Lee: Performed the experiments.

Sami ul Qudoos: Analyzed and interpreted the data.

## Data availability statement

Data associated with this study has been deposited at http://www.med.harvard.edu/aanlib/home.html.

## Declaration of competing interest

The authors declare that they have no known competing financial interests or personal relationships that could have appeared to influence the work reported in this paper.

## Supporting materials

[S_Link 1] https://github.com/yuliu316316/NSST-PAPCNN-Fusion.

[S_Link 2] https://github.com/sagar448/Keras-Convolutional-Neural-Network-Python.

[S_Link 3] https://github.com/uzeful/IFCNN.

[S_Link 4] https://github.com/yuliu316316/CSMCA-Fusion.
